# Research on Underwater Complex Scene SLAM Algorithm Based on Image Enhancement

**DOI:** 10.3390/s22218517

**Published:** 2022-11-05

**Authors:** Renhan Wu, Yuzhuo Gao

**Affiliations:** School of Information Engineering, Ningxia University, Yinchuan 750021, China

**Keywords:** visual-inertial SLAM, underwater detection, feature extraction, feature tracking, marginalization

## Abstract

Underwater images typically suffer from less explicit feature point information and more redundant information due to wild conditions. To solve these degradation problems, we propose the VINS-MONO algorithm to enhance the quality of the underwater image. Specifically, we first used the FAST feature point extraction algorithm to improve the extraction speed. Then, the inverse optical flow method was used to improve the accuracy of feature extraction. At the same time, several kinds of residual information were extracted and marginalized, separately, in the marginalization part of the back-end, in order to improve the marginalization speed. Extensive experiments on underwater dataset HAUD-Dataset and public dataset EuRoC show that our approach is superior to the original VINS-MONO algorithm. In addition, the original algorithm optimizes the situation in which the feature point information is not obvious, and the redundant information is more complex in the underwater environment, which effectively improves the visual quality of the underwater image.

## 1. Introduction

With the rapid development of artificial intelligence, sensors, and other fields such as the field of mobile robots, which combine these fields, have seen rapid developments. Simultaneous Localization and Mapping (SLAM) has also become a necessary technology for mobile robots. Visual SLAM, with the camera alone, is not very effective in practical applications. Visual inertia SLAM with an Inertial Measurement Unit (IMU) overcomes the shortcomings of visual SLAM.

In the Visual Inertial Odometer (VIO), VINS-MONO is a relatively mature algorithm that has been well studied [1,2,3,4]. Although the VINS-MONO algorithm has a relatively good performance in the actual scene operation effect, and is one of the best algorithms in the current SLAM algorithm of vision-inertia fusion, there are still some shortcomings in its underwater use. In the VINS-MONO algorithm, the Harris corner feature extraction algorithm [5], KLT optical flow feature tracking algorithm [6], and IMU pre-integration algorithm, are used in initialization, marginalization, loopback detection, etc. In this study’s aim to resolve the shortcomings of the feature point information in the underwater complex environment, I propose the optimization feature extraction algorithm, feature tracking algorithm, and marginalization, to improve the underwater performance of the VINS-MONO algorithm. The first areas that need to be improved in the underwater environment are:

1. The KLT optical flow method is adopted for tracking and matching, so the robustness and accuracy are poor for the environment, with weak texture and few key points.
2. The Corner extraction algorithm adopts Harris corner. The algorithm adopts Gaussian filtering, which makes the corner extraction speed slow.
3. Marginalization: Several types of residual information are put together for marginalization and optimization, which is costly.

According to the deficiency of the feature tracking algorithm in the first point, this paper will use the inverse optical flow method to improve the accuracy of tracking and matching key points. According to the deficiency of the second algorithm, this paper uses the FAST algorithm [7] to replace the Harris algorithm, in order to accelerate the speed of corner extraction. According to the shortcomings of the third algorithm, this paper separates several types of residual information, which is embodied in the retention and optimization of several types of residual information, by using different strategies. The feasibility of the results is embodied in reading and displaying the video data set information, the running speed of the algorithm on public dataset EuRoC [8] and underwater dataset HAUD-Dataset [9], the extraction speed of printing each algorithm corner point, the recognition accuracy of comparing feature points, the acceleration of the marginalization of VINS-MONO at the back end, and the analysis of detection results. Based on the existing data set, comparing the original VINS-MONO algorithm with the improved one, and comparing the corner point extraction speed, feature point recognition accuracy, and marginalization speed, prove that the algorithm is optimized.

In this paper, we improve the accuracy of the VINS-MONO algorithm, the speed of feature extraction, and accelerate the marginalization. In the second section of this paper, the important work of VINS-MONO algorithm optimization is introduced. The third section introduces the optimization method of the VINS-MONO algorithm. The fourth section is the comparison experiment and results between the original algorithm and the improved algorithm. The fifth section is the conclusion.

## 2. Related Work

Tong et al. [1] proposed a tightly coupled visual-inertial SLAM algorithm and VINS-MONO algorithm, based on optimization. Meanwhile, the author of the paper [1] added closed-loop detection and global optimization to the algorithm. Through the experiments of the VINS-MONO algorithm and the effects mentioned in the paper, it can be proved that the VNIS-MONO algorithm is more stable and more accurate than OKVIS algorithm [10] in most data sets.

In 2019, Shan et al. [11] proposed that the limitations of the fusion monocular camera were changed to the RGBD camera in order to increase the observation information, which solved the problem of unobservability in the original algorithm and was called VINS-RGBD. In 2020, Zhao, HF et al. [12] discussed the application of VINS-MONO in some underwater environments, because the FAST corner feature extraction method adopted by VINS-MONO may lead to the generation of a large number of cycle candidate points, and feature matching may have mismatches, there is not enough loop in the underwater environment. The robustness of the outliers is improved by using Dark Channel Prior (DCP) to enhance the image. For the method proposed by VINS-MONO, more loops can be detected. In 2021, M. He and R. R. Rajkumar et al. [13] extended VINS-MONO, using GNSS and other absolute positioning methods, as well as relative positioning methods based on the Kalman filter. The optimized Extended VINS-MONO algorithm (Extended VINS-MONO) has better accuracy and more accurate positioning.

In 2021, Y. Wang, J. Wang et al. [14] proposed the use of a constant filling method to solve the problem of missing image edges in the FAST corner detection algorithm, and to shorten the corner detection time. In 2020, H. Zhang et al. [15] combined FAST corner detection with LK pyramid optical flow, which could not only quickly detect feature points, but also improve the accuracy of sub-pixel calculation. Mao et al. [16] proposed a double threshold algorithm to solve the threshold setting problem in the optimization of the Harris algorithm. M. Zhao et al. [17] proposed an adaptive parameter algorithm, based on the Harris algorithm, to solve the problem of inaccurate corner detection caused by fixed Gaussian parameters. S. Han et al. [18] used the B-spline function, instead of the Gaussian window function, to improve the accuracy of corner points, as well as pre-selection of diagonal points to improve the real-time performance of the algorithm. Liu Zhen bin et al. [19] improved the initialization based on the VINS-MONO algorithm and added acceleration bias to the initialization optimization algorithm. In 2021, M. He and R. R. Rajkumar et al., according to the optimized VINS-MONO algorithm (Extended VINS-MONO) [13], proposed to add a thermal imager sensor into the algorithm. When the spectral camera gives a poor performance in poor lighting conditions, the thermal vision provided by the thermal imager can make up for these shortcomings [20]. In 2019, L. J. Chen et al. [21] used the VINS-MONO algorithm to test the UAV in a room without GPS signal. In the experiment, the UAV, capable of self-positioning, was constructed by integrating the onboard computer, camera and IMU. A comparison study is given to determine the robustness and reliability of the VINS-Mono state estimator and the UAV system, using various flight velocities and environment features settings. In 2018, TD Chen, H. Jian et al. [22] proposed an image pyramid to track fast-moving targets. within comparison to the dense optical flow method and the color feature method, the results show that the proposed method has many advantages, for example, less computation, better coping with occlusions, and detecting and tracking fast moving objects. Although the pyramid LK optical flow [23] method can deal with large motion, it has the problem of accuracy. In 2018, Z. Wang et al. [24] improved optical flow tracking accuracy by layering the video of each frame in the image pyramid, calculating the optical flow in the top corner, using the next pyramid as the starting point of the pyramid, and repeating this process until the bottom pyramid image.

## 3. Proposed Method

**Figure 1:**
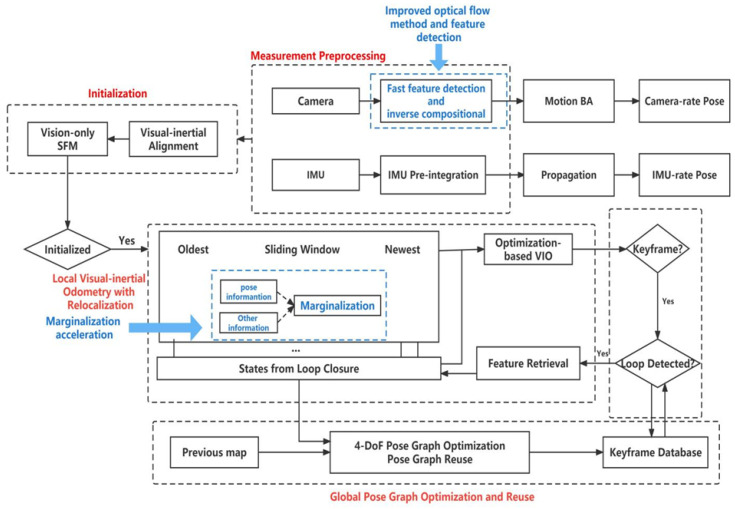
VINS-MONO process structure diagram. The blue dotted box and blue font are the improvements of VINS-MONO algorithm in this paper.

In this paper, we introduce the Harris corner feature extraction algorithm with the FAST corner feature extraction algorithm, and the inverse optical flow method with KLT optical flow and back-end marginalization acceleration, which is shown in Figure 1. Specifically, we accelerate the marginalization by separating the marginalization of pose and information other than pose. The accuracy and speed of the VINS-MONO algorithm are improved from these three aspects.

### 3.1. FAST Corners and Harris Corners

The FAST corner point primarily uses the local image pixel grayscale difference to detect points of interest, and can do so quickly. The corner points extracted by FAST are selected based on the intensity of the pixels around the candidate feature points. For example, in the case of a circle, if the intensity of the pixels on the circle is significantly different from the intensity of the pixels in the center of the circle, then that is a key point. According to experience, a circle with a radius of three can obtain better results and improve the calculation efficiency when selecting key points. If there are more than 12 of the 16 points on the circle, and the gray value of the central point is greater than the threshold, it is a candidate corner point, and the optimal corner point is selected by non-maximum suppression. Non-maximum suppression generally selects the corner with the largest gray difference between the center of the circle and adjacent nodes, and then retains it as the best corner.

The Harris corner is a feature extraction algorithm based on gray image, which adopts Gaussian filtering and has a slow operation speed. The principle is that corner points have large horizontal and vertical gradients, while edge points have large horizontal or vertical gradients, and other points have small horizontal and vertical gradients. Therefore, once the gradient is computed, the corner point can be determined, based on the constraint.

The Harris feature detection method uses a small window, near the feature point, to observe the change of intensity value in a certain direction in the window. Assuming displacement (u,v), then covariance can be used to represent strength change:

(1) $$R=\sum^{\;}{\left(I\left(x+u,y+v\right)-I\left(x,y\right)\right)}^{2}$$

Therefore, the steps of Harris feature detection should be as follows: First, measure the direction where the average strength value changes most obviously, and then measure whether the strength value changes greatly in the vertical direction; if it has, it is an angular point.

The above process can be approximated by Taylor’s formula expansion and verified:

(2) $$R\approx \sum^{\;}{\left((I\left(x,y\right)+\frac{\partial I}{\partial x}u+\frac{\partial I}{\partial y}v-I\left(x,y\right)\right)}^{2}=\sum^{\;}\left({\left(\frac{\partial I}{\partial x}u\right)}^{2}+\frac{\partial I}{\partial y}v^{2}+2\frac{\partial I}{\partial x}\frac{\partial I}{\partial y}\mathrm{uv}\right)$$

The matrix form is:

(3) $$R\approx \left[u,v\right]\left[\begin{array}{cc} \sum^{\;}{\left(\frac{\delta I}{\delta x}\right)}^{2} & \sum^{\;}\left(\frac{\delta I}{\delta x}\frac{\delta I}{\delta y}\right)\\ \sum^{\;}\left(\frac{\delta I}{\delta x}-\frac{\delta I}{\delta y}\right) & \sum^{\;}{\left(\frac{\delta I}{\delta y}\right)}^{2} \end{array}\right]\left[\begin{array}{c} u\\ v \end{array}\right]$$

By calculating the eigenvalues of the verifiable matrix:

(4) $$\mathrm{Dst}\left(x,y\right)=\mathrm{Det}\left(C^{\left(x,y\right)}\right)-k\cdot {\left({\mathrm{trC}}^{\left(x,y\right)}\right)}^{2}$$

Set the parameter K to adjust the performance of the results; K is taken as (0.05–0.5). The parameter K is a constant, and is just a coefficient of the function, and it exists only to regulate the shape of the function

According to the above description, the Harris corner feature extraction method adopts Gaussian filtering in order to slow the feature extraction speed, whilst the FAST corner feature extraction method can effectively compensate for the problem of extraction speed, and gives a better performance in real-time environments.

### 3.2. Optical Flow and Inverse Optical Flow

The LK optical flow method is the representative of the sparse optical flow, in which there is a premise assumption, the gray invariant assumption: the gray value of the same spatial point in each image is unchanged.

At time (t), the gray level of the pixel at (x,y) (x and y are the corresponding pixel coordinate positions in the window) can be written as:

(5) I(x,y,t)

When the pixel moves to (x+dx,y+dy) at t+dt, based on the assumption that the pixel gray value remains unchanged, the following equation can be obtained:

(6) I(x+dx,y+dy,t+dt)=I(x,y,t)

Expand the first order Taylor term on the left:

(7) $$I\left(x+\mathrm{dx},y+\mathrm{dy},t+\mathrm{dt}\right)\approx I\left(x,y,t\right)+\frac{\partial I}{\partial x}\mathrm{dx}+\frac{\partial I}{\partial y}\mathrm{dy}+\frac{\partial I}{\partial t}\mathrm{dt}$$

Since the gray level is assumed to be unchanged, the following equation can be obtained:

(8) $$\frac{\partial I}{\partial x}\mathrm{dx}+\frac{\partial I}{\partial y}\mathrm{dy}+\frac{\partial I}{\partial t}\mathrm{dt}=0$$

Reduction to

(9) $$\frac{\partial I}{\partial x}\frac{\mathrm{dx}}{\mathrm{dt}}+\frac{\partial I}{\partial y}\frac{\mathrm{dy}}{\mathrm{dt}}=-\frac{\partial I}{\partial t}$$

So dx/dt is u, dy/dt is v, ∂I/∂x is $I_{X}$, is $I_{y}$, change in time is $I_{t}$.Writing it in matrix form:

(10) $$\left[I_{x}{\;I}_{y}\right]\left[\begin{array}{c} u\\ v \end{array}\right]=-I_{t}$$

Can know the matrix:

(11) $$A=\left[\begin{array}{c} {\left[I_{x},I_{y}\right]}_{1}\\ .\\ .\\ {\left[I_{x},I_{y}\right]}_{k} \end{array}\right],b=\left[\begin{array}{c} I_{t1}\\ .\\ .\\ I_{\mathrm{tk}} \end{array}\right]$$

we get the equation:

(12) $${\left[\begin{array}{c} u\\ v \end{array}\right]}^{*}=-{\left(A^{T}A\right)}^{-1}A^{T}b$$

The motion velocity u and v of pixels between images can be obtained through calculation.

**Figure 2:**
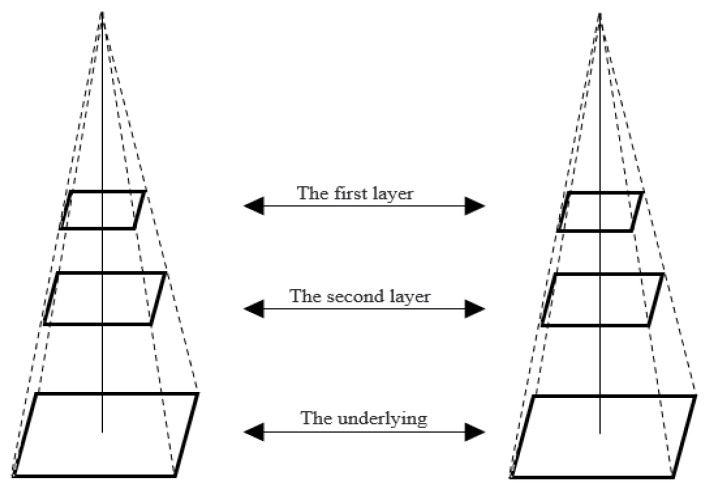
Pyramid flow.

When the camera moves too fast, the direct calculation of the single-layer optical flow may cause local extreme values due to excessive changes. It is necessary to scale the image through pyramid optical flow to improve this situation. Take the original image as the bottom layer of the pyramid and scale the image one layer up to achieve a pyramid shape, as shown in Figure 2.

The optical flow method computes the H matrix $\left(H=J\cdot J^{T}\right)$ (J is the Jacobian matrix) through the least square method at each iteration, which causes a large amount of calculation; while the inverse optical flow method is the inverse of the forward optical flow, and the forward optical flow changes the direction. Optical flow covers the range from the feature point of an image (denoted as X) to a different position in the next image (denoted as Y), as the camera moves, while the inverse optical flow is from the image Y to the image X; that is, from the Y after the motion to the X before the motion. In inverse optical flow, since X is the image before motion and does not move, the H matrix has no relation to movement. However, H is constant when calculating the increment of movement in each iteration. The H matrix only needs to be calculated once, in the first iteration, which greatly reduces the amount of calculation.

### 3.3. Marginalization Acceleration

If changes to the camera pose is calculated only from the two frames, it is fast, but with low accuracy. However, if the global optimization method (such as Bundle Adjustment [25]) is adopted, the accuracy is high, but the efficiency is low. Therefore, the sliding window method is introduced, which optimizes a fixed number of frames at a time. This ensures both accuracy and efficiency. Since it is a sliding window, new image frames will come in, and old image frames will leave, in the process of sliding. The process of marginalization is designed to make good use of the image frames that remain. Marginalization is designed to delete some useless pictures, but retain the information used in the image, such as prior information, IMU information, etc. Marginalization converts them into prior information, which is encapsulated and then added into nonlinear optimization. It is assumed that the state to be marginalized is x2, and the state to be retained is *x*1. For the incremental equation Hδx=b, become:

(13) $$\left[\begin{array}{cc} H_{11} & H_{12}\\ H_{21} & H_{22} \end{array}\right]\left[\begin{array}{c} \delta_{x_{1}}\\ \delta_{x_{2}} \end{array}\right]=\left[\begin{array}{c} b_{1}\\ b_{2} \end{array}\right]$$

The marginalization method is the Schur complement matrix

(14) $$\left[\begin{array}{cc} I & -H_{12}H_{22}^{-1}\\ 0 & I \end{array}\right]\left[\begin{array}{cc} H_{11} & H_{12}\\ H_{21} & H_{22} \end{array}\right]\left[\begin{array}{c} \delta_{x_{1}}\\ \delta_{x_{2}} \end{array}\right]=\left[\begin{array}{cc} I & -H_{12}H_{22}^{-1}\\ 0 & I \end{array}\right]\left[\begin{array}{c} b_{1}\\ b_{2} \end{array}\right]$$

solve the equation:

(15) $$\left(H_{11}-H_{12}H_{22}^{-1}H_{21}\right)\delta_{x_{1}}=b_{1}-H_{12}H_{22}^{-1}b_{2}$$

The original incremental equation is derived as follows:

(16) $$H_{0}^{*}\delta_{x}=J_{l}^{T}J_{l}\delta_{x}=b^{*}=b_{0}^{*}+H_{0}^{*}\mathrm{dx}=b_{0}^{*}+J_{l}^{T}J_{l}\mathrm{dx}=J_{l}^{T}{\left(J_{l}^{T}\right)}^{+}b_{0}^{*}+J_{l}^{T}J_{l}\mathrm{dx}=J_{l}^{T}\left({\left(J_{l}^{T}\right)}^{+}b_{0}^{*}+J_{l}\mathrm{dx}\right)$$

Equivalent prior error after marginalization is:

(17) $$e_{p}={\left(J_{l}^{T}\right)}^{+}b^{*}={\left(J_{l}^{T}\right)}^{+}b_{0}^{*}+J_{l}\mathrm{dx}$$

The aim of marginalization acceleration is to first marginalize the parts, with the exception of the camera pose, and then to marginalize the camera pose. The reason for this two-step process is the marginalization of the camera pose, because the amount of difference is too large, two separate threads can play a role in acceleration.

## 4. Experiments

In this paper, we compare the VINS-MONO algorithm with the improved VINS-MONO algorithm in the public dataset EuRoC and the underwater dataset HAUD-Dataset. By printing the feature tracking speed and marginalization speed, and using the EVO trajectory measurement tool, it is more intuitive to see where the algorithm has been improved. EVO is a trajectory assessment tool for visual odometry and SLAM problems. The core functionality is the ability to plot the trajectory of the camera or evaluate the error of the estimated trajectory from the true value. The absolute pose error (APE), often used as the absolute trajectory error, compares the estimated trajectory with the reference trajectory and calculates the statistics of the entire trajectory, which is suitable for testing the global consistency of the trajectory. The relative pose error (RPE) does not compare the absolute pose, but the relative pose error compares the motion (attitude increment). The relative pose error can give the local accuracy. Furthermore, the relative pose error (RPE) is divided into translation error and rotation error.

**Figure 3:**
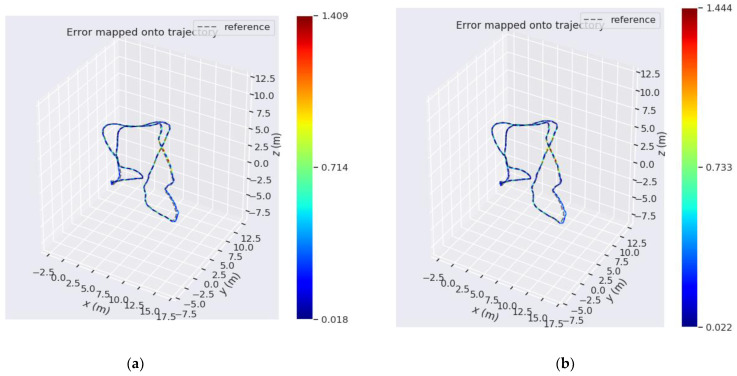
(**a**) is the trajectory error of VINS-MONO algorithm with loop detection on MH_05_difficult data set. (**b**) Trajectory error of loopback detection on MH_05_difficult data set for optimized VINS-MONO. (The unit of scale is m).

**Figure 4:**
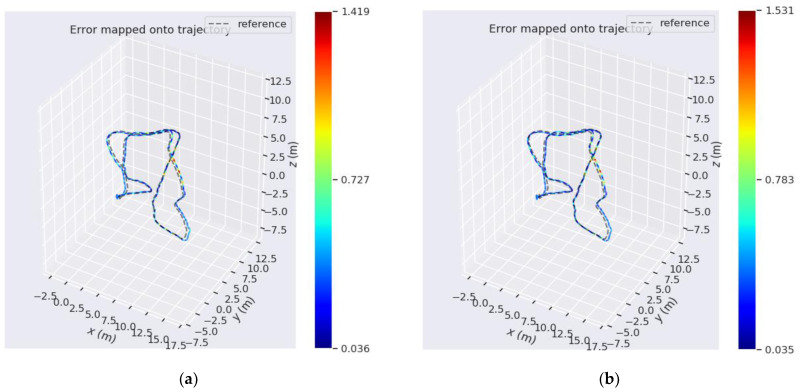
(**a**) is the trajectory error of VINS-MONO algorithm in MH_05_difficult data set without loopback detection. (**b**) is the trajectory error of the optimized VINS-MONO without loopback detection on MH_05_difficult data set. (The unit of scale is m).

**Figure 5:**
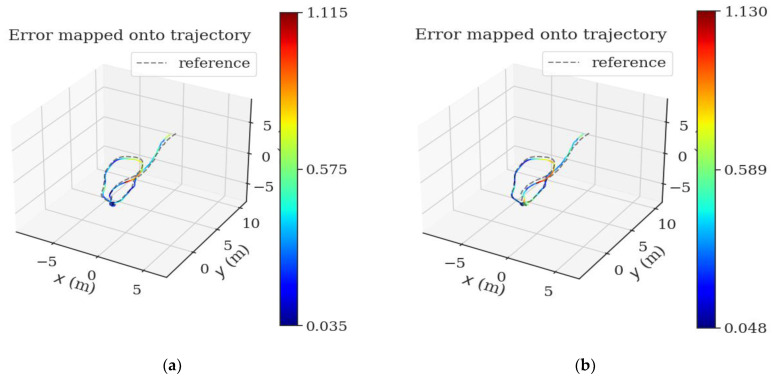
(**a**) is the trajectory error of VINS-MONO algorithm on sequence_03.bag dataset. (**b**) is the trajectory error of the optimized VINS-MONO on Sequence_03.bag dataset.

Taking MH_05_difficult dataset in EuRoC dataset as an example to compare track errors, as shown in Figure 3 and Figure 4:

Secondly, the sequence_03.bag dataset in the underwater HAUD-Dataset is taken as an example to compare the trajectory errors, as shown in Figure 5:

### 4.1. Accuracy Comparison of the Algorithms in Public Dataset EuRoC

The KLT pyramid optical flow tracking algorithm has poor robustness and accuracy for the environment, with a weak texture and few key points. Therefore, this paper adopts the inverse optical flow method to replace the KLT pyramid optical flow feature tracking algorithm that is used in the original algorithm in order to improve the accuracy of the algorithm. Two algorithms were used to run all of the data sets provided by EuRoC dataset and Root-Mean-Square Error (RMSE) was used to compare the accuracy of the VINS-MONO algorithm with the optimized VINS-MONO algorithm. As shown in Table 1, Table 2, Table 3 and Table 4.

### 4.2. Comparison of the Algorithm’s Corner Extraction Speed in the Public Dataset EuRoC

In terms of the feature extraction speed, the Harris corner extraction algorithm adopts Gaussian filtering to reduce the speed of corner extraction. Therefore, the FAST corner extraction algorithm is adopted in this paper to replace the Harris corner extraction algorithm in order to improve the speed of feature extraction. The speed of the original algorithm and the improved algorithm is compared through the results of operation in EuRoC dataset, as shown in Table 5.

### 4.3. The Algorithm Compares the Back-End Marginalization Speed in the Public Dataset EuRoC

To accelerate marginalization, the algorithm prints out the marginalization time, and compares the marginalization time between the original algorithm and the improved algorithm by running EuRoC data set to reflect the acceleration of marginalization, as shown in Table 6.

### 4.4. Accuracy Comparison and Speed Comparison of Algorithms in Underwater HAUD-Dataset

**Figure 6:**
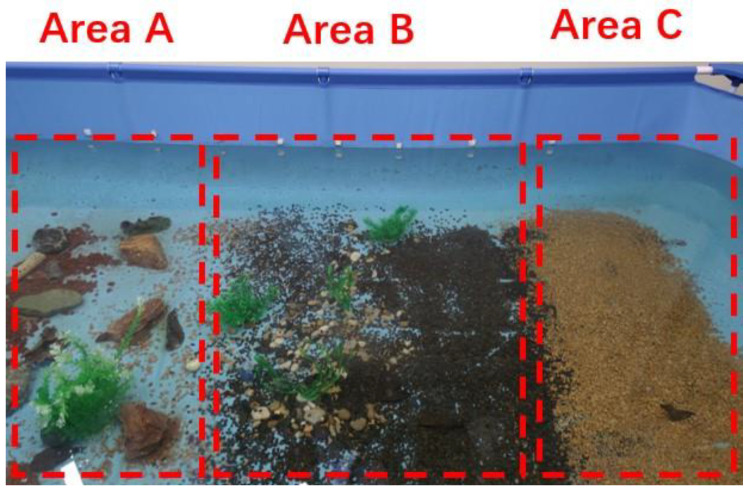
There are three areas in the underwater data acquisition pool, and different areas contain different levels of texture [9].

Figure 6 shows the experimental scene of the underwater data set. The KLT pyramid optical flow tracking algorithm has poor robustness and accuracy for the environment, with a weak texture and few key points. Therefore, this paper adopts the inverse optical flow method to replace the KLT pyramid optical flow feature tracking algorithm in the original algorithm to improve the accuracy of the algorithm. In terms of the feature extraction speed, the Harris corner extraction algorithm adopts Gaussian filtering to reduce the speed of corner extraction. Therefore, the FAST corner extraction algorithm is adopted in this paper to replace Harris corner extraction algorithm, in order to improve the speed of feature extraction. The underwater data sets sequence_03.bag, sequence_05.bag, sequence_06.bag, sequence_07.bag were selected, and the VINS-MONO algorithm and the optimized algorithm, were used to run these four data sets and compare the accuracy (as shown in Table 7), feature point extraction speed (as shown in Table 8) and marginalization speed (as shown in Table 9). Thus, the accuracy and speed of the algorithm are greatly improved after optimization. In addition, the accuracy of the optimized algorithm and the VINS-Fusion algorithm are compared in these four data sets (as shown in Table 10).

**Figure 7:**
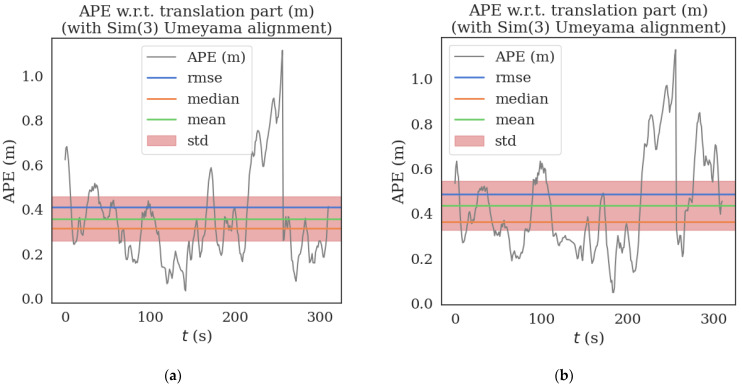
(**a**) is the Absolute Pose Error (APE) of the improved algorithm and (**b**) is the Absolute Pose Error (APE) of the original algorithm. The black line is the absolute pose error (APE), the blue line is the root mean square error (RMSE), the orange line is the median error (median), the green line is the mean error (mean), and the red area is the standard deviation (std).

**Figure 8:**
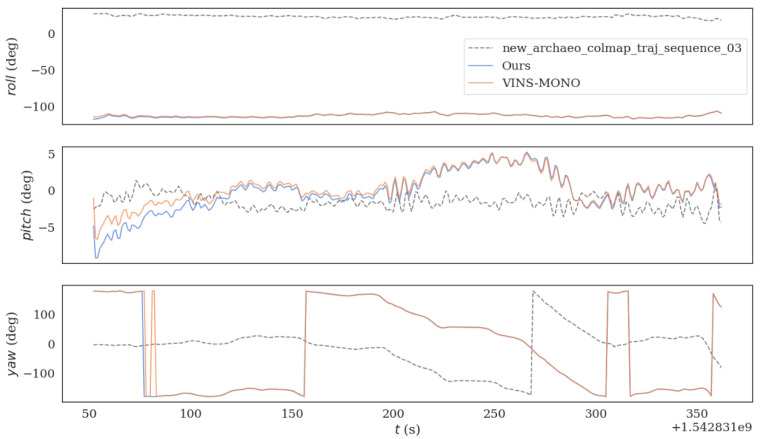
The figure shows the rotation error comparison between the original algorithm and the improved algorithm in sequence_03.bag datasets.

**Figure 9:**
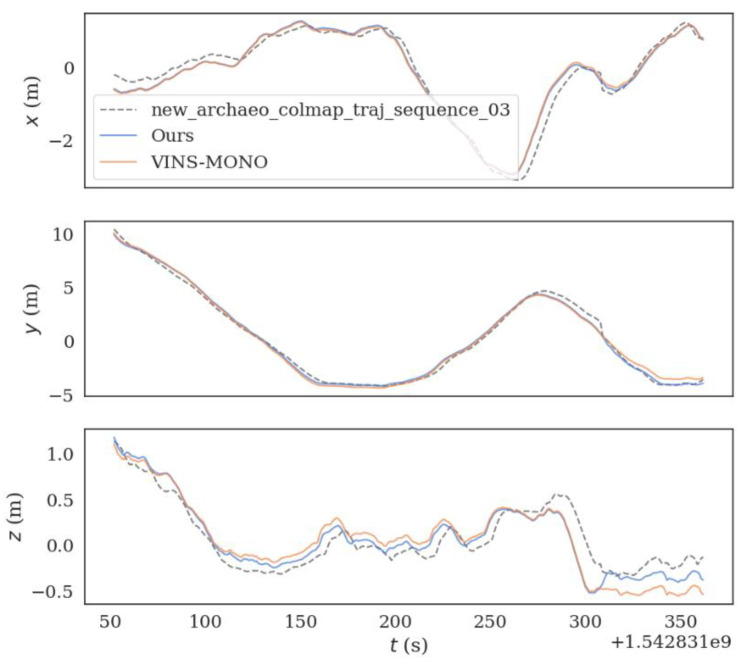
The figure shows the translation error comparison between the original algorithm and the improved algorithm in sequence_03.bag datasets.

The two algorithms were run on sequence_03.bag datasets provided in HAUD-Dataset, and RMSE, rotation error and translation error were used to compare the accuracy of the optimized VINS-MONO algorithm and VINS-MONO algorithm, as shown in Figure 7, Figure 8 and Figure 9.

**Figure 10:**
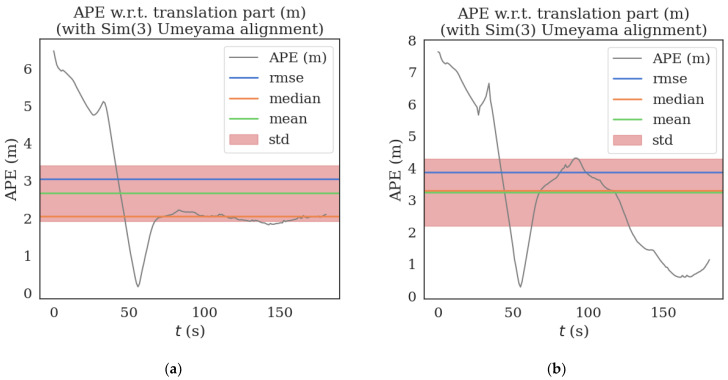
(**a**) is the Absolute Pose Error (APE) of the improved algorithm and (**b**) is the Absolute Pose Error (APE) of the original algorithm. The black line is the absolute pose error (APE), the blue line is the root mean square error (RMSE), the orange line is the median error (median), the green line is the mean error(mean), and the red area is the standard deviation (std).

**Figure 11:**
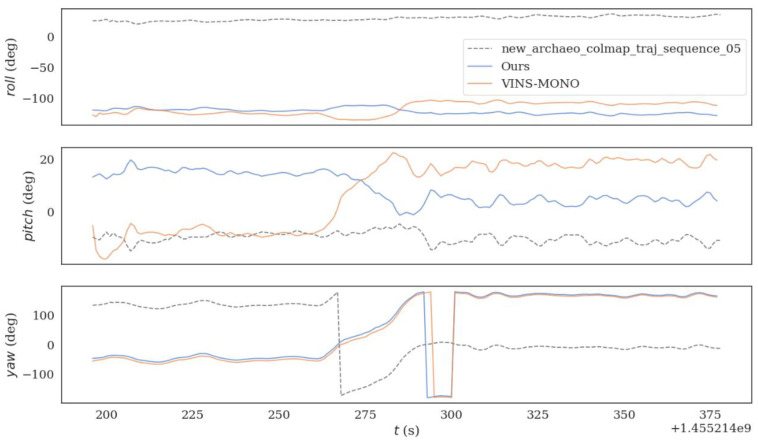
The figure shows the rotation error comparison between the original algorithm and the improved algorithm sequence_05.bag datasets.

**Figure 12:**
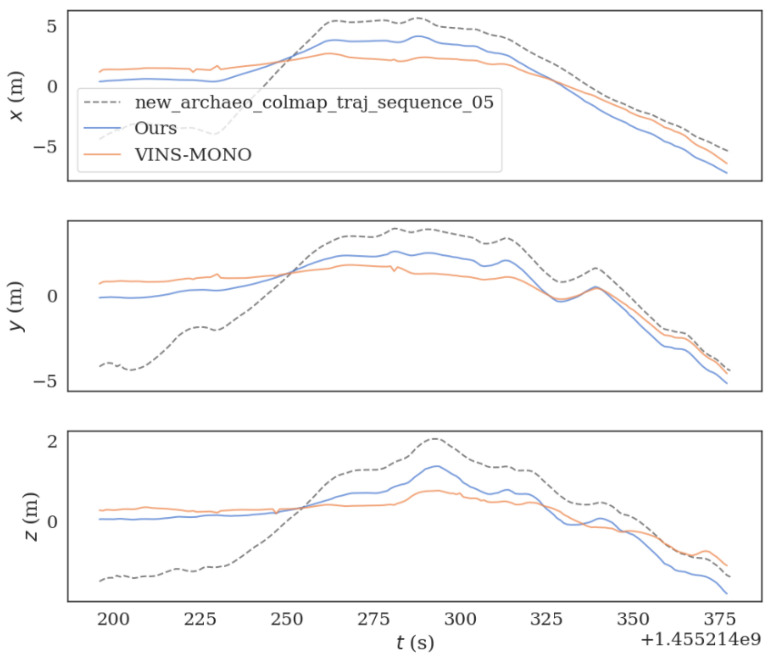
The figure shows the translation error comparison between the original algorithm and the improved algorithm in sequence_05.bag datasets.

The two algorithms were run on sequence_05.bag datasets provided in HAUD-Dataset, and RMSE, rotation error and translation error were used to compare the accuracy of the optimized VINS-MONO algorithm and VINS-MONO algorithm, as shown in Figure 10, Figure 11 and Figure 12.

## 5. Discussion and Analysis

The comparison experiment, between the VINS-MONO algorithm and the optimization algorithm, was carried out on the open dataset EuRoC and the underwater dataset HAUD-Dataset. Firstly, the accuracy of the original algorithm was compared with the improved algorithm in the open dataset EuRoC (as shown in Table 11 and Table 12). The accuracy of the optimized algorithm is higher than that of the original algorithm under the condition that most EuRoC data are concentrated without loopback, and the overall accuracy is improved by 0.8 percent. The accuracy of the optimized algorithm is 0.2 percent higher than that of the original algorithm under the condition of loopback.

According to the data in the table, it can be concluded that the use of inverse optical flow significantly increases the number of effective matching points and eliminates some miscellaneous points during triangulation, therefore improving the accuracy. In addition, when optimized by the MH_04_difficult data set, the accuracy of the algorithm in dark scenes and fast movement is significantly improved, with a nine percent increase under the condition of a loop-back. 

In the open dataset EuRoC, the original algorithm and the improved algorithm are compared in terms of feature extraction speed (as shown in Table 13), and the overall average time is shortened by 1.5 ms.

According to the data in the table, it can be concluded that using the FAST feature extraction algorithm to replace the Harris feature extraction algorithm can improve the speed of feature extraction and shorten the extraction time.

Finally, the marginalization time of the optimized algorithm is shortened by 2384 ms, on average, compared with the original algorithm, as shown in Table 14.

By comparing the accuracy of the original algorithm and the optimized algorithm with the HAUD-Dataset (as shown in Table 15) with a weak texture and fewer key points, the overall accuracy is improved by 4.2 percent.

According to the comparison of accuracy data between the original algorithm and the optimized algorithm in the underwater data set, it can be concluded that the optimized algorithm has higher accuracy and is more suitable for underwater complex scenes.

In the underwater dataset HAUD-Dataset, the original algorithm and the improved algorithm are compared in terms of feature extraction speed, and the overall average time is shortened by 1.0 ms, as shown in Table 16.

According to the comparison of feature point extraction speed, it can be concluded that the optimized algorithm is faster in the underwater complex situation.

In the underwater dataset HAUD-Dataset, the marginalization time of the optimized algorithm is shortened by 5892 ms on average compared with the original algorithm, as shown in Table 17.

Comparing the accuracy of the optimized algorithm with the VINS-Fusion algorithm in the public dataset EuRoC and the underwater dataset HAUD-Dataset, it can be concluded that the accuracy of the optimized algorithm is improved by 1.6% and 3.75%, respectively, as shown in Table 18 and Table 19.

From the analysis of the experimental data, it can be concluded that on the open dataset EuRoC and the underwater dataset HAUD-Dataset, the optimized algorithm is superior to the original algorithm in terms of accuracy, feature point extraction speed and marginalization speed.

## 6. Conclusions

The VINS-MONO algorithm has a good performance in vision-inertial SLAM, however, there are still some shortcomings in the feature extraction speed and recognition accuracy in the underwater complex environment. The purpose of this study is to solve the current shortcomings of the VINS-MONO algorithm and put forward solutions to optimize the VINS-MONO algorithm, as well as comparative tests to verify the feasibility of the solution.

In this paper, the first measure of optimization of VINS-MONO is to optimize the feature extraction speed. The FAST corner feature extraction algorithm is used to replace the Harris corner feature extraction algorithm, which makes up for the disadvantage of slow feature extraction speed, so that the VINS-MONO algorithm has a significant improvement in feature extraction speed. The second measure is that we use an inverse optical flow method, rather than forward optical flow, which improves the recognition accuracy of the algorithm and greatly reduces the amount of calculation. The third measure is to optimize several types of residual information for the back-end marginalized part. In the original algorithm, these types of residual information were marginalized and optimized together, while in this paper, different strategies were used to reserve and optimize different residual information, thus improving the speed of marginalization.

In this paper, the public dataset EuRoC and the underwater dataset HAUD-Dataset are used for comparative experiments, which show that the optimized algorithm offers a good improvement in feature extraction speed, recognition accuracy and marginalization speed. In the future, we will compare each visual-inertial SLAM algorithm with the VINS-MONO algorithm in all aspects, find out the shortcomings of VINS-MONO algorithm, and optimize it. At the same time, we will use the optimized algorithm for practical application research, find out the existing problems in the algorithm, and optimize. Furthermore, in future experiments, the optimized algorithm will be applied to more scenes, from which the shortcomings of the algorithm under more constraints are found. According to these shortcomings, possible solutions are proposed, and the algorithm is further studied.

## Figures and Tables

**Table 1:** The Absolute Pose Error (APE) of the original algorithm is compared with that of the improved algorithm in the EuRoC dataset. (Unit: meter).

	Original VINS-Loop	Original VINS-Noloop	Improved VINS-Loop	Improved VINS-Noloop
MH_01_easy	0.18	0.27	0.18	0.25
MH_02_easy	0.18	0.23	0.18	0.22
MH_03_medium	0.40	0.43	0.40	0.42
MH_04_difficult	0.39	0.50	0.38	0.41
MH_05_difficult	0.38	0.41	0.38	0.41
V1_01_easy	0.14	0.16	0.14	0.16
V1_02_medium	0.31	0.32	0.30	0.31
V1_03_difficult	0.31	0.31	0.31	0.31
V2_01_easy	0.12	0.14	0.12	0.14
V2_02_medium	0.27	0.27	0.27	0.27
V2_03_difficult	0.32	0.43	0.32	0.48

**Table 2:** The absolute pose error of VINS-Fusion algorithm is compared with that of the improved algorithm in the EuRoC dataset. (Unit: meter).

	VINS-Fusion	Improved VINS-MONO
MH_01_easy	0.15	0.11
MH_02_easy	0.16	0.16
MH_03_medium	0.39	0.35
MH_04_difficult	0.37	0.34
MH_05_difficult	0.32	0.32
V1_01_easy	0.13	0.13
V1_02_medium	0.28	0.26
V1_03_difficult	0.31	0.31
V2_01_easy	0.12	0.12
V2_02_medium	0.27	0.22
V2_03_difficult	0.32	0.32

**Table 3:** The translation error of the original algorithm is compared with that of the improved algorithm. (Unit: meter).

	Original VINS-Loop	Original VINS-Noloop	Improved VINS-Loop	Improved VINS-Noloop
MH_01_easy	0.19	0.15	0.19	0.15
MH_02_easy	0.20	0.16	0.20	0.16
MH_03_medium	0.42	0.36	0.42	0.36
MH_04_difficult	0.40	0.34	0.40	0.34
MH_05_difficult	0.39	0.32	0.38	0.32
V1_01_easy	0.15	0.13	0.15	0.13
V1_02_medium	0.30	0.30	0.30	0.30
V1_03_difficult	0.23	0.22	0.23	0.22
V2_01_easy	0.12	0.10	0.11	0.10
V2_02_medium	0.24	0.23	0.24	0.23
V2_03_difficult	0.25	0.25	0.25	0.25

**Table 4:** The rotation error of the original algorithm is compared with that of the improved algorithm. (Unit-less).

	Original VINS-Loop	Original VINS-Noloop	Improved VINS-Loop	Improved VINS-Noloop
MH_01_easy	0.09	0.07	0.09	0.07
MH_02_easy	0.09	0.07	0.09	0.07
MH_03_medium	0.13	0.11	0.13	0.11
MH_04_difficult	0.10	0.08	0.10	0.08
MH_05_difficult	0.10	0.08	0.10	0.08
V1_01_easy	0.13	0.12	0.13	0.12
V1_02_medium	0.24	0.24	0.24	0.24
V1_03_difficult	0.24	0.23	0.24	0.23
V2_01_easy	0.13	0.11	0.13	0.11
V2_02_medium	0.21	0.20	0.21	0.20
V2_03_difficult	0.21	0.21	0.21	0.21

**Table 5:** The feature extraction speed of the original algorithm is compared with that of the improved algorithm in the EuRoC dataset. (Unit: ms).

	Original VINS	Improved VINS
MH_01_easy	5.29	6.08
MH_02_easy	7.60	5.18
MH_03_medium	9.28	5.31
MH_04_difficult	6.86	5.72
MH_05_difficult	7.94	5.62
V1_01_easy	3.19	5.79
V1_02_medium	7.11	4.84
V1_03_difficult	3.04	3.33
V2_01_easy	3.64	6.28
V2_02_medium	4.99	4.85
V2_03_difficult	16.13	6.05

**Table 6:** Compare the marginalization speed between the original algorithm and the improved algorithm in the EuRoC dataset. (Unit: ms).

	Original VINS	Improved VINS
MH_01_easy	16,533.17	10,500.04
MH_02_easy	10,642.57	7370.98
MH_03_medium	12,259.10	9193.03
MH_04_difficult	6802.97	5700.21
MH_05_difficult	8606.01	6153.79
V1_01_easy	12,726.89	10,519.55
V1_02_medium	5698.64	3893.45
V1_03_difficult	5131.97	3342.73
V2_01_easy	8735.87	7433.12
V2_02_medium	7408.65	5503.44
V2_03_difficult	4429.29	3129.24

**Table 7:** The Absolute Pose Error (APE) of the original algorithm is compared with that of the improved algorithm in the underwater dataset. (Unit: meter).

	Original VINS	Improved VINS
sequence_03.bag	0.5	0.41
sequence_05.bag	3.9	3.1
sequence_06.bag	1.5	1.5
sequence_07.bag	5.8	5.8

**Table 8:** The feature extraction speed of the original algorithm is compared with that of the improved algorithm in the underwater dataset. (Unit: ms).

	Original VINS	Improved VINS
sequence_03.bag	9.34	7.71
sequence_05.bag	11.25	8.12
sequence_06.bag	8.72	8.39
sequence_07.bag	13.36	14.52

**Table 9:** Compare the marginalization speed between the original algorithm and the improved algorithm in the underwater dataset. (Unit: ms).

	Original VINS	Improved VINS
sequence_03.bag	15,806.17	9450.34
sequence_05.bag	18,463.48	11,621.76
sequence_06.bag	14,684.65	8563.45
sequence_07.bag	9423.42	5169.47

**Table 10:** The absolute pose error of VINS-Fusion algorithm is compared with that of the improved algorithm in the underwater dataset. (Unit: meter).

	VINS-Fusion	Improved VINS-MONO
sequence_03.bag	0.4	0.35
sequence_05.bag	3.2	3.2
sequence_06.bag	1.1	1.1
sequence_07.bag	4.9	4.8

**Table 11:** Percentage of accuracy improvement between the original algorithm and the optimized algorithm without loopback (unit: meter).

	Original VINS-Noloop	Improved VINS-Noloop	The Percentage
MH_01_easy	0.27	0.25	2
MH_02_easy	0.23	0.22	1
MH_03_medium	0.43	0.42	1
MH_04_difficult	0.50	0.41	9
MH_05_difficult	0.41	0.41	0
V1_01_easy	0.16	0.16	0
V1_02_medium	0.32	0.31	1
V1_03_difficult	0.31	0.31	0
V2_01_easy	0.14	0.14	0
V2_02_medium	0.27	0.27	0
V2_03_difficult	0.43	0.48	−5
Average value	\	\	0.8

**Table 12:** Percentage of accuracy improvement between the original algorithm and the optimized algorithm with loopback (unit: meter).

	Original VINS-Loop	Improved VINS-Loop	The Percentage
MH_01_easy	0.18	0.18	0
MH_02_easy	0.18	0.18	0
MH_03_medium	0.40	0.40	0
MH_04_difficult	0.39	0.38	1
MH_05_difficult	0.38	0.38	0
V1_01_easy	0.14	0.14	0
V1_02_medium	0.31	0.30	1
V1_03_difficult	0.31	0.31	0
V2_01_easy	0.12	0.12	0
V2_02_medium	0.27	0.27	0
V2_03_difficult	0.32	0.32	0
Average value	\	\	0.2

**Table 13:** Speed comparison between the original algorithm and the optimized algorithm (unit: ms).

	Original VINS	Improved VINS	Difference Value
MH_01_easy	5.29	6.08	−0.79
MH_02_easy	7.60	5.18	2.42
MH_03_medium	9.28	5.31	3.97
MH_04_difficult	6.86	5.72	1.14
MH_05_difficult	7.94	5.62	2.32
V1_01_easy	3.19	5.79	−2.6
V1_02_medium	7.11	4.84	2.27
V1_03_difficult	3.04	3.33	−0.29
V2_01_easy	3.64	6.28	−2.64
V2_02_medium	4.99	4.85	0.14
V2_03_difficult	16.13	6.05	10.08
Average value	\	\	1.5

**Table 14:** Comparison of the marginalization speed between the original algorithm and the optimized algorithm in the EuRoC dataset (unit: ms).

	Original VINS	Improved VINS	Difference Value
MH_01_easy	16,533.17	10,500.04	6033
MH_02_easy	10,642.57	7370.98	3272
MH_03_medium	12,259.10	9193.03	3066
MH_04_difficult	6802.97	5700.21	1102
MH_05_difficult	8606.01	6153.79	2453
V1_01_easy	12,726.89	10,519.55	2207
V1_02_medium	5698.64	3893.45	1805
V1_03_difficult	5131.97	3342.73	1789
V2_01_easy	8735.87	7433.12	1302
V2_02_medium	7408.65	5503.44	1905
V2_03_difficult	4429.29	3129.24	1300
Average value	\	\	2384

**Table 15:** Percentage of accuracy improvement between the original algorithm and the optimized algorithm in the underwater dataset (unit: meter).

	Original VINS	Improved VINS	The Percentage
sequence_03.bag	0.5	0.41	9
sequence_05.bag	3.9	3.1	8
sequence_06.bag	1.5	1.5	0
sequence_07.bag	5.8	5.8	0
Average value	\	\	4.2

**Table 16:** The feature extraction speed of the original algorithm and the improved algorithm is compared in the underwater dataset (Unit: ms).

	Original VINS	Improved VINS	Difference Value
sequence_03.bag	9.34	7.71	1.63
sequence_05.bag	11.25	8.12	3.13
sequence_06.bag	8.72	8.39	0.33
sequence_07.bag	13.36	14.52	−1.16
Average value	\	\	1.0

**Table 17:** Compare the marginalization speed between the original algorithm and the optimized algorithm in the underwater dataset (Unit: ms).

	Original VINS	Improved VINS	Difference Value
sequence_03.bag	15,806.17	9450.34	6355
sequence_05.bag	18,463.48	11,621.76	6841
sequence_06.bag	14,684.65	8563.45	6121
sequence_07.bag	9423.42	5169.47	4253
Average value	\	\	5892

**Table 18:** Compare the accuracy of VINS -Fusion algorithm with the improved algorithm in the EuRoC dataset (Unit: meter).

	VINS-Fusion	Improved VINS-MONO	The Percentage
MH_01_easy	0.15	0.11	4
MH_02_easy	0.16	0.16	0
MH_03_medium	0.39	0.35	4
MH_04_difficult	0.37	0.34	3
MH_05_difficult	0.32	0.32	0
V1_01_easy	0.13	0.13	0
V1_02_medium	0.28	0.26	2
V1_03_difficult	0.31	0.31	0
V2_01_easy	0.12	0.12	0
V2_02_medium	0.27	0.22	5
V2_03_difficult	0.32	0.32	0
Average value			1.6

**Table 19:** The absolute pose error of VINS-Fusion algorithm is compared with that of the improved algorithm in the underwater dataset (Unit: meter).

	VINS-Fusion	Improved VINS-MONO	The Percentage
sequence_03.bag	0.4	0.35	5
sequence_05.bag	3.2	3.2	0
sequence_06.bag	1.1	1.1	0
sequence_07.bag	4.9	4.8	10
Average value	/	/	3.75

## Data Availability

https://projects.asl.ethz.ch/datasets/doku.php?id=kmavvisualinertialdatasets, https://bat.sjtu.edu.cn/zh/haud-dataset/, accessed on 20 August 2022.
